# On Dentition and Some Coincident Disorders

**Published:** 1842-06

**Authors:** 


### Bibliographical Notices.

On Dentition *and some coincident Disorders.* By John Ashburner, M. D. Member of the Royal College of Physicians, &c. &c. London, 1834, pp. 235, 18mo.

We do not recollect to have ever read a book of so small a size that contained so much really useful information as the one of which the above is the title. It is a most excellent little treatise, and though we do not believe all the doctrines advocated in it to be correct, yet nevertheless, its excellencies are so much greater and more numerous, than what we conceive to be its faults, we do not hesitate to pronounce it one of the very best works of the kind with which we are acquainted. Dr. Ashburner is an able and ingenious physiologist, and a good writer. He possesses in an eminent degree, the happy ability of conveying his ideas in a forcible manner, in few words. What he says, is generally to the point.

The work has been before the profession in England for some years, and we would be glad to see it republished in this country. It would no doubt meet with a ready sale both from the medical and dental professions. But our object more especially, in noticing it at this late period, is to direct the attention of our own more immediate professional brethren to it. The information contained in it will richly compensate for the trouble of its procurement and perusal.

From the following extracts the readers will be able to judge of the author's ability, and form some idea of the value of the work.—*Balt. Ed.*

"There is no circumstance relating to growth more striking, or more curious, than the division of life into epochs. The climacteric years of the ancients were multiples of seven; and they were pretty accurate observers of the changes which took place in the body at different periods. That there must be a rule regulating the grand epochs of development in a *perfectly healthy* individual, there can be no doubt. In the course of life, man appears, in the changes to which his frame is subjected, to go through several types of configuration: the same individual that had once, in the womb of his parent, the shape of a worm, and that subsequently rapidly traversed the types of other gradations of the lower animals, and became an infant breathing the surrounding air, is by no means to be recognized as *identical* with the vigorous man of thirty-five. His physiognomy alone points out the changes effected upon him, and we cannot doubt that there must be some ground for the vulgar observation that has come down to us, of the body undergoing remarkable changes during the course of successive climacterics. His progress through his varied types of configuration is dependent on the completion of each stage of his course. The measurement of time by chronometer—the indications of a completion of each advance of period—are illustrative of the trains through various successive types in the course of the development of a perfectly healthy person. The circumstances relating to dentition are but as isolated set of phenomena. All the organs of the body should have their chronicles; but it will be found that they are the wheels and springs of the time-piece. The chief *index* is remarked in the organs of mastication, and they respond to epochs.

When the first dentition is complete, nature appears to rest. The teeth, in number and in size, being sufficient for the wants of the child, there seems for a time to be no further effort of growth among them. The principle of a continual progress is, however, established in the system. Though there is an apparent rest, there is, in fact, a real march in all parts of the body. The clock does not incessantly strike, but the hands are moving. The law which regulates the trains of growth throughout the body, insists upon an additional energy being communicated at certain periods to organs whose epochs of advancement to a type of more exalted perfection had arrived. It is not to be supposed that because the first set of teeth have all appeared, there is no change in progress in the jaws, or in the germs of the second set of teeth. The jaws are gradually expanding, and as they are becoming larger, the capsules of the teeth of the second dentition are preparing the organs they have to build for assuming their stations.

Let us consider the changes undergone by the jaws and by the teeth of the first dentition. When the progress of growth has extended so far as to render the first set of twenty teeth insufficient for the purposes of complete mastication, they appear to become loose in their sockets: in reality, an absorptive process is established, and their roots are eaten away. The pulp having first decayed, the crown crumbles away from the gums. Normally, this change takes place in the teeth exactly in the order in which they were originally formed, and in which they cut through the gums. The central incisors of the lower jaw fall away first; then those of the upper jaw, between which the growth of the bone has created a very remarkable space; then go the interior lateral incisors; and so on. Before, however, any of the teeth of the first set fall out, the jaw has already acquired a considerable growth, and four molar teeth have appeared at the back of the existing twenty teeth. These four are permanent teeth; they occupy a large space, and their relations to the first set and to the teeth which are afterwards to appear, will be more advantageously explained after some further anatomical inquiries; and these must relate to the organs concerned in supplying the first and second set of teeth with nourishment, and the history of the changes they undergo, in order that the deciduous teeth, and the processes of bone which belong to them, may vanish, and allow the permanent set to grow up and take a normal alignement."

"How do the teeth of the second dentition proceed to displace those of the first dentition? Much absurdity has been published on this question. They do not displace the teeth of the first dentition. The jaws grow; they enlarge: as their volume increases, the germs of the permanent teeth continue to develope the organs they have to form. These germs are enclosed in cells in the bony substance of the jaws. Up to the age of five, six, or seven years, the jaws of a child may be said to contain two sets of *sockets.* Hunter, Blake, De la Barre, and Serres, use this expression, though it is not quite correct; for the teeth of the second dentition, though not yet perfect, are lodged in cells below, and a little behind, the alveolar processes of the jaw which belong to the first teeth. The cells of the second teeth occupy a curve less extensive than the alveoli of the first, and are separated from these by a bony lamina, forming a kind of partition, which keeps the two sets of teeth quite distinct from each other.

The germs of the second set of teeth have long existed in the jaws. It has been remarked, that the germs of the first dentition are attached, in the fœtus, immediately to the membranous folds, which, at this period, constitute the gums; and that those of the second dentition are suspended from them by means of small pedicles. When the capsules of the first dentition were advancing towards their development, and were approaching the upper part of the gum, those of the second dentition appeared to retreat into the depth of the jaw, and hung to the gums by their pedicles. The pedicle, in the progress of growth, is destined to perform an important part. It becomes a fibrous canal, communicating between the alveolar margin and the cell in which the capsule is lodged: it is apparently periosteum; but whatever may be its real nature, it leads to the tooth, and becomes continuous with the external layer of the dental membrane."

"We see very few persons who have not suffered more or less severely in the mouth, from the local consequences of anormal dentition. It would be very trite to speak of the common occurrence of tooth-ache; yet the subject of the influence of anormal growth in the production of diseases of the mouth, has been very little investigated. It is generally acknowledged as a fact, that children suffer more or less during the progress of dentition; and that very few adults are seen with a perfectly normal condition of the mouth; indeed, so rare is the occurrence of a normal state of the teeth, that the beauty of a fine set of teeth is a striking object of admiration. The local inconveniences resulting from anormal trains of growth in the teeth and jaws are not few. The surgeon is already well acquainted with this part of our subject. Among them may be enumerated those congenital defects more or less allied to the fissure we know as hare-lip; irregularity in the curved alignement of the teeth; decayed teeth; other diseased states of the teeth inflammations, abscesses, and tumors, resulting from these, or from irritations in the course of growth to the jaw-bones, muscular, cellular, and glandular tissues of these parts. The mouth, the throat, the nostrils, the palate, and all the neighbouring parts, have disease inflicted upon them occasionally, by anormal conditions of the teeth and jaws."

"If the proposition had at any time been put forth, that anormal states of dentition were invariably and immediately the *causes* of serious disorders of health, a doctrine would have been advanced which the experience of most medical men would have enabled them to subvert at once. The country practitioner more especially would instantly put his negative upon the assertion; for in his experience, unless the district in which he practises be a marshy country, or have circumstances about it depressive of the vital forces, anormalities in development are, for the most part, compensated by the energy which a pure and invigorating atmosphere communicates to the nervous system. In the country, obstacles to the normal progress of growth in the teeth and jaws, may, and occasionally do, intervene; but the forces of the constitution are in most cases sufficient to overcome the inconveniences resulting from them. In London, and in large towns, it is different; errors in the progress of growth communicate their influence to various organs; and according to the tendencies of the constitution, according to the *idiosyncrasies* of the individual, or according to the condition in which surrounding circumstances may have for the time placed him, the mischief, which may vary certainly in degree, is felt in one set of organs or in another. Unless much more were known of the precise coincidence which a certain anormality may require—of the precise trains of symptoms indicating certain erroneous trains of growth—it would be impossible to point out the diseased states and irregular developments that are linked with each other.

We are not aware fully of the coincident events connected with development, which take place in the earlier periods of infancy—a time of life when the mobility of the frame, and the rapidity of change in the course of growth, render their consequences striking. In pointing out, therefore, the links in the chains of diseases which hang on irregularities in the course of development, the most that can at present be attempted is to trace analogies; and even in this task, the complications that beset every event in the animal economy may tend to confound us, and to lead us wide of the direct line of our inquiry.

Regarding the infant, we ask if coincidences of disease with anormal dentition can fairly be traced before the usual period of cutting the teeth? Superficial observers have a fashion of scouting ideas inconsistent with the notions they have imbibed from their masters. We know well that children, at very early periods after birth, are the subject of convulsions. Irritations upon the skin may be vicarious with these epileptic attacks, or irritations upon the mucous part of the alimentary canal may affect the nervous centre. Why may not a turgid state of vessels about the dental matrices, in the simpler and less complete conditions of organization belonging to this period of life, immediately compress the dental, or even the maxillary nerves? The subject of convulsions in earlier infancy is yet involved in considerable obscurity, and if we shall at a future time see reason to approach the conclusion, that pressure applied under certain conditions to a nervous tendril of a motor portion of the fifth pair is nearly tantamount to pressure applied to certain parts of the brain, we may well believe that irregularities in the course of growth at the earlier periods of infant life may produce pressure that shall be succeeded by convulsions. Perhaps pressure or other irritation, applied to a sensitive nerve, may be succeeded tic douloureux, or a slighter neuralgia.

A fine healthy-looking child of a strong Irish woman died at thirteen weeks of age, of a convulsion fit. My suspicions as to its having had improper food were not well founded. The mother was anxious to have the cause of death ascertained, and I found no difficulty in obtaining leave to open the body. The organs for the most part were healthy; the stomach contained only a little milk; no error upon the mucous surfaces of the intestines. Skin perfectly healthy. The contents of the thorax, as well as those of the abdomen, were quite healthy. In the head there was a slightly injected state of the vessels of the pia mater, but in other respects the brain was quite healthy. The capsules of the incisor teeth were large and very vascular, much more advanced than usual. With a lancet the cartilaginous rim of the lower jaw was attempted to be removed, with a view of exposing the capules of the molar teeth; but these were so unusually distended with fluid, that the instrument cut into them and let it out. This was an example of development proceeding too hastily."

